# Kinetics of humoral deficiency in CART19-treated children and young adults with acute lymphoblastic leukaemia

**DOI:** 10.1038/s41409-020-01027-6

**Published:** 2020-08-14

**Authors:** A. Deyà-Martínez, A. Alonso-Saladrigues, A. P. García, A. Faura, M. Torrebadell, A. Vlagea, A. Català, A. Esteve-Solé, M. Juan, S. Rives, L. Alsina

**Affiliations:** 1 Clinical Immunology and Primary Immunodeficiencies Unit, Allergy and Clinical Immunology Department, Hospital Sant Joan de Déu, Universitat de Barcelona, Pediatric Research Institute Sant Joan de Déu, Barcelona, Spain; 2 Clinical Immunology Unit, Hospital Sant Joan de Déu-Hospital Clínic, Universitat de Barcelona, Barcelona, Spain; 3 CAR T-Cell Unit, Department of Pediatric Hematology and Oncology, Hospital Sant Joan de Déu, University of Barcelona, Pediatric Research Institute Sant Joan de Déu, Barcelona, Spain; 4 Department of Immunology-CDB, Hospital Clínic-IDIBAPS, Universitat de Barcelona, Barcelona, Spain; 5 Immunotherapy Platform, Hospital Sant Joan de Déu-Hospital Clínic, Universitat de Barcelona, Barcelona, Spain

**Keywords:** Paediatrics, Acute lymphocytic leukaemia

## Abstract

CD19-CAR T-cell therapy (CART19) causes B-cell aplasia (BCA) and dysgammaglobulinemia but there is a lack of information about the degree of its secondary immunodeficiency. We conducted a prospective study in children and young adults with acute lymphoblastic leukaemia treated with CART19, analysing the kinetics of BCA and dysgammaglobulinemia during therapy, as well as the B-cell reconstitution in those with CART19 loss. Thirty-four patients were included (14 female) with a median age at CART19 infusion of 8.7 years (2.9–24.9). Median follow-up after infusion was 7.1 months (0.5–42). BCA was observed 7 days after infusion (3–8), with persistence at 24 months in 60% of patients. All patients developed a progressive decrease in IgM and IgA: 71% had undetectable IgM levels at 71 days (41–99) and 13% undetectable IgA levels at 185 days (11–308). Three of 12 patients had protective levels of IgA in saliva. In two of three patients who lost CART19, persistent B-cell dysfunction was observed. No severe infections occurred. In conclusion, BCA occurs soon after CART19 infusion, with a progressive decrease in IgM and IgA, and with less impairment of IgA, suggesting the possibility of an immune reservoir. A persistent B-cell dysfunction might persist after CART19 loss in this population.

## Introduction

Chimeric antigen receptor (CAR) T-cell therapy uses genetic engineering to express a surface receptor against a tumour antigen on T-cells, combining, in a chimeric molecule, the antigen specificity of an antibody, with signalling domains allowing cytotoxic antitumoral function [1, 2].

The most extensively investigated of these receptors is CAR19 T-cell therapy (CART19), used in B-cell malignancy treatment [1–3] with very promising results. To date, two CAR-T cell constructs have been approved by both the Food and Drug Administration and the European Medicines Agency for two different indications: the treatment of relapsed refractory B-cell acute lymphoblastic leukaemia (ALL) in children and young adults (Tisagenlecleucel, Kymriah®), and for the treatment of refractory or relapsed diffuse large B-cell lymphoma in adults (Tisagenlecleucel, Kymriah® and axicabtagen leucel, Yescarta®) [4–9].

CART19 therapy causes B-cell aplasia (BCA) of variable duration. CD19 is expressed on the surface of B-cells during all maturation stages (from pro-B cell in bone marrow to memory B cell and plasmablasts). All CD19-positive B-cells are targeted by CART19 therapy and destroyed. There are currently two recognised subsets of antibody-secreting cells [10]: plasmablasts and plasma cells. CD19 is expressed in plasmablasts but not in plasma cells [11, 12]. The absence of plasma cells has been linked to more severe forms of primary antibody deficiencies, such as congenital agammaglobulinemia; these patients are more prone to developing infectious complications and require immunoglobulin replacement therapy (IgRT) [13].

Secondary to CD19+ B-cell destruction, patients develop a progressive decrease in all immunoglobulin isotypes (IgG, IgA and IgM): some in a form of dysgammaglobulinemia (deficiency of one or more, but not all, classes of immunoglobulins) and others in the form of agammaglobulinemia (extremely low level of all classes of immunoglobulins). Only sporadic reports [1, 14, 15] have been published analysing the degree of CART19-induced secondary immune-deficiency in paediatric patients, and current recommendations on IgRT in CART19-treated paediatric patients are extrapolated from primary agammaglobulinemia [15, 16], because they share the BCA. Nevertheless, patients may not be entirely comparable because of expected plasma cell persistence after CART19 therapy, which has recently been confirmed [17].

Likewise, only sporadic reports have described B-cell recovery once CAR T-cell becomes non-functional, as well as the timing for IgRT withdrawal [15]. Thus, there is currently a need to gain insight into the type and degree of secondary antibody deficiency caused by CART19 therapy in paediatric patients, in order to optimise IgRT in order to avoid infectious complications.

For this purpose, we analysed, within a single-centre cohort of patients with ALL, the kinetics of BCA and dysgammaglobulinemia during CAR19 T-cell therapy, and the pattern of B-cell reconstitution in those patients in whom CART19 lost its function.

## Methods

This was a prospective study, developed in a single centre, between January 2016 and October 2019, which included children and young adults, from 0 to 25 years old, with ALL that received lentiviral CAR19 T-cell therapy with 4–1BB- and CD3z-signalling domains with two different constructs: CTL019/tisagenlecleucel (EudraCT 2016–001991–31 [7] and post-marketing use) and ARI0001 cells (EUDRACT 2016–002972–29) [18]. The main difference between these two constructs is the peptide sequence from the single chain variable region of the monoclonal antibody against CD19 (scFV), which in ARI-cells is A3B1 [18] and in CTL09 is FCM63 [7].

Patients received lymphodepleting therapy (Fludarabine 90–120 mg/m^2^ and Cyclophosphamide 900–1000 mg/m^2^) 1 week prior to CAR T-cell infusion. Thereafter, all patients received IgRT starting at day 28 after CART19 infusion, (monthly, 0.5 g/kg/dose; trough levels > 8 g/L), along with antibiotic prophylaxis (Protocol available in Supplementary Table S1), irrespective of infections and baseline IgG levels, according to current recommendations for paediatric population receiving CART19 [15], which differ from those of adults.

**Fig. 1:**
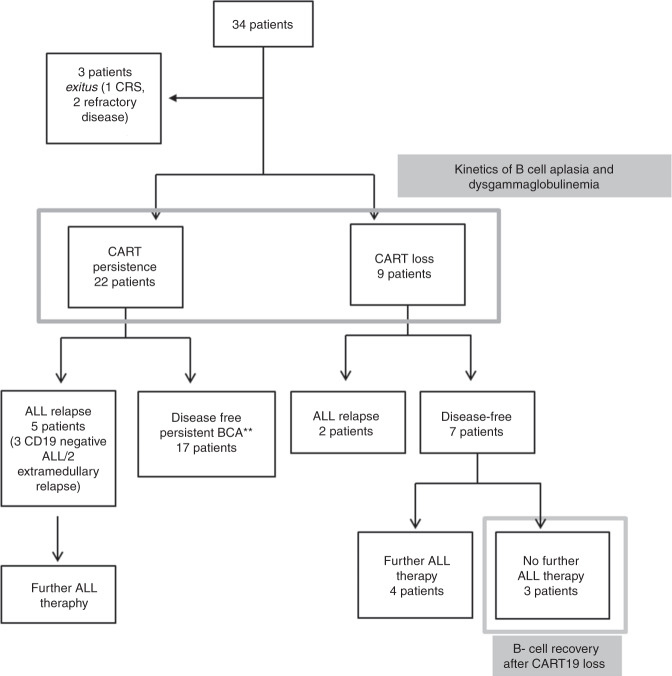
CART19-cohort description. Cohort included children and young adults, from 0 to 25 years old, with ALL that received lentiviral CAR19 T-cell therapy with 4–1BB- and CD3zsignalling domains with two different constructs: CTL019/tisagenlecleucel (EudraCT 2016–001991–31) and ARI0001 cells (EUDRACT 2016–002972–29). The kinetics of BCA and dysgammaglobulinemia were analysed every 1–3 months in 31 patients, and after B-cell recovery in 3 patients (grey boxes).

Humoral immunity was analysed every 1–3 months both during BCA (*n* = 34) and after B-cell recovery (*n* = 3, Fig. 1) and included immunoglobulin levels of IgG, IgM and IgA (in serum and saliva, age-related normal values) (ARCHITECT Systems® and AEROSET System. Immunoturbidimetric measure); CD4+ T-cells, CD8+ T-cells, CD19+ B-cells, B-cell immunophenotyping including I cells and memory CD27+ cells (Flow Cytometry Analysis, Becton Dickinson FACS Canto®).

In order to rule out the presence of T-cell dysfunction in relation to prior ALL treatment, T-cell function was evaluated at least once, 6–12 months after CART19 infusion, by testing lymphocyte proliferative responses to mitogens (PHA, ConA, PWM).

The evaluation of the persistence or loss of vaccine-induced antibody titres after CART19 infusion was not feasible because all patients received IgRT as early as 28 days after infusion, and the administered polyclonal immunoglobulin products contain an array of antigen-specific IgG that would yield positive results to any serologic testing performed while on IgRT. In patients under IgRT, only polysaccharide responses to *Salmonella tiphy* can be measured [19], which is a T-independent response (relying on B cells), and thus not assessable due to CART19-induced BCA.

In those patients who had CART19 loss and no further therapy (e.g HSCT) during the B-cell reconstitution period, IgRT withdrawal was guided by the increase/normalisation of IgM/IgA and the patient’s ability to develop an appropriate antigen-specific response. For the latter, *S. tiphy* vaccine responses were used, as mentioned above [19].

Loss of CART19 function was defined as B cells being detectable in peripheral blood (Flow cytometry. Detection threshold >50 B-cells/mm^3^) [20]. Cytokine release syndrome (CRS) was graded according to the 2019 ASBMT consensus [21]. Complete response, refractoriness or relapsed disease under CAR19 T-cell therapy was defined according to standard ALL criteria (See Supplementary Table S2**)**.

Patient information was collected from the medical chart. Patients with prior diagnosis of a primary immunodeficiency were excluded from the analysis. The study was reviewed and approved by the ethics committee of our institution.

### Statistical analysis

Categorical and continuous variables were described as percentages and median values with ranges (min–max). For the comparative analysis, Chi-square test and Mann-Whitney U test were applied, as appropriate, for the data set. Pearson and Spearman tests were used to identify correlations between quantitative variables. The analysis was carried out using SPSS version 15.0 software (SPSS Inc., Chicago, IL), and statistical significance was set at *p* ≤ 0.05.

## Results

### Baseline characteristics of the studied cohort

A total of 34 patients received CAR19 T-cell therapy during the study period: 23 with CTL019 (CTL019/Tisagenlecleucel) and 11 with ARI 0001 cells (Table 1).

**Table 1:** Baseline characteristics of the CART19 patient cohort (*N* = 34).

	*N* = 34
Female	14/34 (51.2%)
Acute lymphocytic leukaemia (ALL)	34/34 (100%)
Age at ALL (years) (median, IQR)	4.2 (0.25–21.5)
Previous HSCT (once/twice)	26/34 (76.5%) (22/26 vs 4/26)
Previous total body irradiation (12–18 Gy)	3/34 (8.8%)
Previous B-cell targeted therapy^a^	9/34 (26.5%) 2: ino/2 rtx/4 blina/1 borte
Prior ALL lines of therapy^b^	
One line	1/34 (2.9%)
Two lines	18/34 (52.9%)
Three lines	9/34 (29.4%)
Four lines	4/34 (11.8%)
Five lines	2/34 (5.9%)
CART19 Type	
CTL019/tisagenlecleucel	23/34 (67.6%)
ARI-0001	11/34 (32.3%)
Age at CART19 therapy (years)	8.7 (2.9–24.9)
Time between ALL diagnosis and CART19 infusion (months) (median, IQR)	48.1 (14.9–139.2)
Decrease in immunoglobulins (IgA, M, G) prior to CART19 and lymphodepletion	22/33 (66.7%)
Isolated IgM	8/33 (24.2%)
IgG + IgM	6/33 (18.2%)
IgA + IgM	3/33 (9.1%)
IgG + IgA + IgM	2/33 (6.1%)
IgG + IgA	1/33 (3%)
Isolated IgA	1/33 (3%)
Isolated IgG	1/33 (3%)
Post CART19-infusion follow up (months)	7.1 (0.5–42)
CART19 loss	10/34 (29.4%)
Patient’s outcome after CART19 infusion	
Mortality^c^	6/34 (17.6%)
Refractory	2/34 (5.9%)
ALL Relapse	7/34 (20.6%)
HSCT after CART19	7/34 (20.6%)
Clinical and Immunological data after CART19 infusion^d^	*N* = 31
CRS after infusion	23/32 (71.9%)
CRS grade III–IV	8/23 (17.4%)
Cytopenia >28 days after infusion^e^	20/30 (66.7%)
Isolated Anaemia	5/20 (25%)
Isolated Neutropenia	1/20 (5%)
Isolated Thrombocytopenia	4/20 (20%)
Two cytopenias	5/20 (25%)
Three cytopenias	5/20 (25%)
Infections during CART19 therapy	
Patients with infections during first 28 days^f^	6/31 (19.3%)
Patients with infections beyond day 28th^g^	7/31 (22.5%)
Decreased IgM levels below normal per age	31/31 (100%)
Undetectable IgM	22/31 (71%)
Time to achieve IgM below normal levels per age (days) (median, IQR)	14 (2–98)
Time to achieve undetectable IgM (days) (median, IQR)	71 (41–99)
Decreased IgA levels below normal per age	31/31 (100%)
Undetectable IgA	4/31 (12.9%)
Time to achieve undetectable IgA (days) (median, IQR)	185 (11–308)
Time to achieve IgA below normal levels (days) (median, IQR)	20 (7–59)
Time to achieve undetectable B cells (days)	7 (1–33)
Abnormal T-cell proliferative responses to mitogens	2/15 (13.3%)
Detectable IgA in saliva	4/12 (33.3%)

*ALL* acute lymphoblastic leukaemia, *IQR* interquartilic range, *CART19* CD19-Chimeric antigen receptor, *HSCT* haematopoietic stem cell transplant, *Gy* grey, *CRS* cytokine release syndrome.

^a^Rituximab (rtx), inotuzumab (ino), blinatumomab (blina), bortezmib (borte).

^b^A line of therapy consists of ≥1 complete cycle of a single agent, a regimen consisting of a combination of several drugs or a planned sequential therapy of various regimens.

^c^One patient died from CRS, five patients from progressive disease.

^d^No follow-up data available for three patients who died early-on after CART19: refractoriness (1), non-responder (1) and grade V CRS (1).

^e^One patient lost CART19 at day +40 after infusion, thus this item is no valuable.

^f^Catheter-related infections 4/6 and viral infections 2/6 (one pulmonary adenovirus infection and one HHV-6 encephalitis).

^g^Severe infections in two patients (one H. influenzae meningitis and one pneumonia) and 4/6 patients with mild gastrointestinal and upper respiratory airway infections, mild herpes zoster and adenovirus reactivation.

All patients were diagnosed with ALL and 41% (14/34) were female. The median age at ALL diagnosis was 4.2 years old (0.25–21.5), and at CART19 infusion it was 8.7 years old (2.9–24.9). Treatments received prior to CART19 having an impact on the immune system were as follows: 26 of 34 (76%) had received a hematopoietic stem cell transplantation (HSCT) (22 of 26 once, 4 of 26 twice), 3 of 34 (9%) total body irradiation, and 9 of 34 (26%) specific B-cell targeted therapies or therapies impacting on B cells (two inotuzumab, two rituximab, four blinatumomab and one bortezomib). Prior to CART19 infusion and lymphodepletion, 22 patients (67%) presented some degree of dysgammaglobulinemia according to normal age-matched range levels, with the main disturbance combinations isolated being hypo-IgM (8 of 22) and hypo-IgG plus hypo-IgM (6 of 22).

### Immunological changes during functional CART19 and B-cell aplasia

During CAR T-cell infusion, 23 of 34 developed CRS (8/23 grade III/IV).

**Fig. 2:**
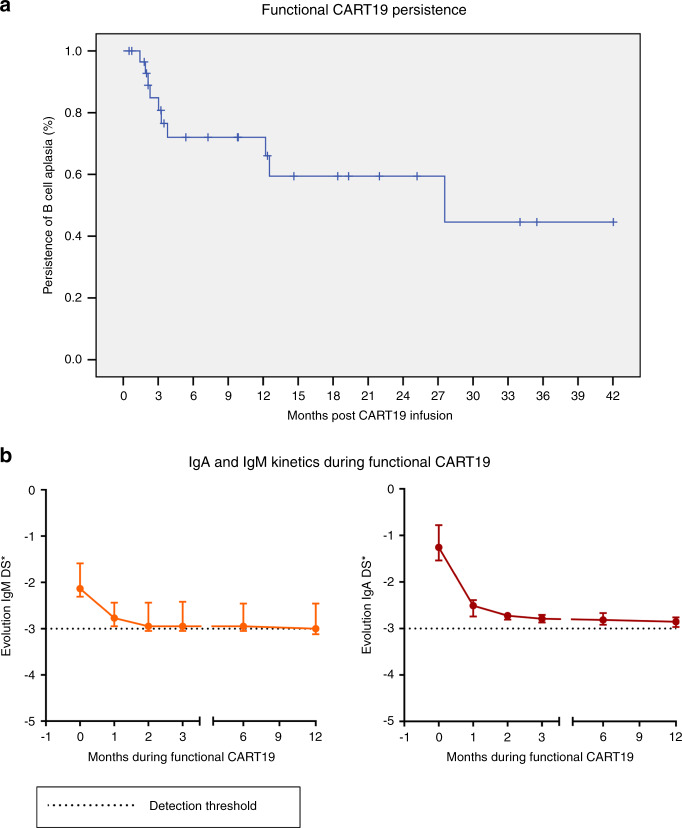
Functional CART19 persistence, and IgA and IgM kinetics during functional CART19. **a** Undetectable B cells (or BCA) were observed 7 days after infusion, with a persistence of BCA at 6, 12, and 24 months in 75%, 60%, and 60% of patients, respectively. **b** After CART19 infusion, all patients developed a progressive decrease in IgM and IgA levels: the first determination of IgM below normal range per age was at day 14 and at day 20 for IgA; 71% progressed to undetectable IgM levels 71 days after infusion, whereas 13% progressed to undetectable IgA levels 185 days after infusion. Immunoglobulin G remained within normal ranges for age under IgRT [32].

Immunological data are available for 31 of 34 patients (Table 1 and Fig. 1). Three patients died and were not assessable: two patients due to CART refractoriness presenting a progressive disease (death at 2 and 9 months, respectively, after receiving CART19) and one patient with severe CRS developed during the first week after CAR T-cell infusion. For the remaining patients, the median follow-up after CART19 infusion was 7.1 months (0.5–42). For all studied patients, undetectable B cells (or BCA) were observed 7 days after infusion (1–33), with a persistence of BCA at 6, 12 and 24 months in 75%, 60%, and 60% of patients, respectively (Fig. 2a). Immunoglobulin G remained within normal ranges for age under IgRT (Supplementary Table S1).

All patients developed a progressive decrease in IgM and IgA levels (Fig. 2b). Regarding IgM, patients reached the first determination below normal range per age at day 14 after CART19 infusion (2–98). Twenty-two patients (71%) progressed to undetectable IgM levels, with a median of 71 days after infusion (41–99). As for IgA, the first determination under normal levels per age-range occurred 20 days after CART19 infusion (7–59), and 4 of 31 (13%) progressed to undetectable levels at 185 days after infusion (11–308).

A test of proliferative lymphocyte response to mitogens was performed in 15 out of 31 patients, being abnormal in two.

A comparative analysis searching for factors related to the development of undetectable IgM or IgA was carried out (Supplementary Table S3). As to undetectable IgM, a significant relation with CART19 persistence was observed (373.5 vs 60 days; p:0.001). No other significant factors were identified. Regarding patients with undetectable IgA, most frequently they were female (9 of 27 vs 4 of 4; p:0.012), had received B-cell targeted therapies prior to CART infusion (5/27 v*s* 3/4, *p* = 0.016) and showed a decrease in IgA levels to below the normal age-matched levels prior to CART19 therapy (3 of 26 vs 2 of 4; p:0.055). No other significant factors were identified.

IgA levels in saliva were analysed in 12 of 31 patients. IgA in saliva was detectable in 4 of 12 patients, being in normal range in 3 of 4 patients. Blood serum IgA levels were detectable in 11 of 12 patients. Only those patients with IgA serum levels above 0.4–0.6 g/L showed detectable IgA saliva levels. Accordingly, a positive and statistically significant correlation between IgA levels in serum and IgA levels in saliva was obtained (correlation 0.79, *p* = 0.002). Patients with detectable IgA in saliva were significantly older at ALL onset (median age 6 vs 2.3 years; *p* = 0.004) and at the time of CART19 infusion (11.6 vs 6.4 years old; *p* = 0.048). No other significant factors were identified.

A total of 6 of 31 patients developed infections during the first 28 days after CART19 infusion, and 7 of 31 beyond this date (but within the period of functional CART19 without relapses).

As for the infections during the first 28 days, four of six were catheter-related infections and two of six viral infections: one rhinovirus causing pulmonary disease (with associated effusion) during CRS, with good evolution within a week, and one VH6 encephalitis (manifested as memory disturbance. MRI was compatible with viral encephalitis, and PCR for VH6 was positive in blood and cerebrospinal fluid. The recovery after foscarnet treatment was complete).

As for the infections beyond day 28, two of seven required admission, one for *H. influenza* meningitis 6 months after CART19 infusion in a patient with prior HSCT and no criteria for amoxicillin prophylaxis, with good evolution under antibiotic treatment, and the other for pneumonia, which evolved without complications, in a patient with prior lung damage (obliterans bronchiolitis post HSCT) and under prophylaxis with trimethoprim-sulfamethoxazole and azithromycin. The other four patients developed minor infections: one gastrointestinal infection, one respiratory tract infection, one herpes zoster, and one one adenovirus reactivation (associated with diarrhoea, with positive detection on blood and stool). All patients had correct IgG trough levels (>8 g/L) at the time of these infections.

### B cell and humoral immunity reconstitution after CART19 loss

Of the 34 patients, 3 presented CART19 loss and were free of disease and did not receive further antileukemic therapy for more than 6 months, permitting evaluation of immune reconstitution.

B-cell recovery of the three patients over a period of 9–27 months is detailed in Table 2. At their last follow-up visit (27, 9 and 22.5 months after CART19 loss, respectively), two of three patients (P1, P3) showed some degree of B-cell impairment, while P2 showed fast and competent B-cell reconstitution.

**Table 2:** Immunological reconstitution after CART19 loss.

	Patient 1	Patient 2	Patient 3
Gender	Female	Male	Female
Age (years)	4.4	18	6.7
Age at ALL diagnosis	5 months	12 years	2 years
Previous HSCT	Yes (11 mo) HSCT with MUD 10/10. PTLD (14 mo) with rtx treatment and low grade chronic cutaneous and gut GVHD	Yes (15.87 yo) HSCT with MRD 10/10. Reduced intensity conditioning regimen	Yes (4 yo). HSCT with MUD 10/10. Conditioning regimen: total corporal irradiation tiotepa and cyclophosphamide
Number of ALL relapses	One	Two	Three
Prior ALL lines of therapy^b^	One line with HSCT after complete remission	Three lines	Four lines
CART19 protocol	CAR T-Novartis® (CTL019-B2202)	CAR T-Novartis® (CCTL019B2001X)	CART ARI0001
CAR T-cell infusion dose	4 × 106/kg CTL019	1.7 × 106/kg CTL019	1st infusion: 5 × 10^6^/kg ARI-0001 2nd infusion^b^: 2 × 10^6^/kg ARI-0001
B-cell aplasia duration (months)	12.2	2.13	9.8
Immunology evaluation at last follow-up visit			
Date of last evaluation after CART19 loss (months)	27	22.5	9
Immunoglobulin levels			
IgG (g/L)	8.66 (6.64–13.7)^a^	6.77 (6.50–15)	12.6 (6.64–13.7)^a^
IgM (g/L)	0.27 (0.45–1.96)	0.56 (0.40–3.45)	0.61 (0.45–1.96)
IgA (g/L)	<0.01 (0.35–1.91)	1.61 (0.76–3.90)	<0.01 (0.35–1.91)
Total B-cell counts (%/abs)	23%/299 (13–27%/270–860)	26%/468 (6–23%/110–570)	24%/216 (13–27%/270–860)
Naïve B-cell counts (%/abs)	98%/258.7 (62–94/70–630)	91.4%/427.75 (33–95%/28–550)	96.7%/208.8 (62–94%/70–630)
Memory CD27+ B cells	0.9%/2.37 (3–18%/7–51)	8.6%/40.24 (5–60%/4.5–130)	1.3%/2.8 (3–18%/7–51)
Total T-cell count (%/abs)	69%/897 (60–76%/1300–2600)	69%/1242 (56–84%/1000–2200)	68%/612 (60–76%/1200–2600)
TL CD4+	42%/546 (31–47%/650–1500)	34%/612 (31–52%/530–1300)	26%/234 (31–47%/650–1500)
TL CD8+	24%/312 (18–35%/370–1200)	32%/576 (18–35%/330–920)	41%/369 (18–35%/370–1200)
Proliferative responses to mitogens	normal	normal	normal
*S. tiphy* antibody responses (U/ml)^b^	Basal: 9.8 Post vaccine: 8.9	Not performed	Not performed
Current status	Free of disease. No HSCT	ALL relapse after 22 months free of disease	HSCT due to myelodysplasia

*ALL* acute lymphoblastic leukaemia, *CART19* CD19-Chimeric antigen receptor, *HSCT* haematopoietic stem cell transplant, *PTLD* post-transplant lymphoproliferative disease, *MUD* matched unrelated donor, *MRD* matched related donor, *rtx* rituximab, *GVHD* graft-versus-host disease, *CTL* cytotoxic T lymphocyte, *TL* T lymphocyte.

^a^Under IgRT **Protective levels 32 U/mL.

^b^A line of therapy consists of ≥1 complete cycle of a single agent, a regimen consisting of a combination of several drugs, or a planned sequential therapy of various regimens.

Patients 1 and 3 did not reach normal age-range absolute B-cell counts (one had normal B cell percentage); B-cells upon reconstitution were mainly composed of naïve B cells (Table 1). Despite B-cell recovery, P1 and P3 showed an impairment of IgA and IgM immunoglobulin production with differing severity, and the patients remain on IgRT. In order to evaluate the capacity of B-cells to develop a polysaccharide-specific response (before attempting to withdraw IgRT), P1 was vaccinated with *Salmonella tiphy*, showing no specific responses post-vaccination (baseline IgG 9.8 U/mL → Post-vaccine response 8.9 U/mL. Protective level >32 U/mL). This vaccination could not be administered to P3 because she developed myelodysplasia and had to proceed to HSCT.

As for patient 2, he presented a fast B-cell recovery, achieving normal B-cell levels for his age 1 month after CART loss, along with progressive recovery of IgA (detectable at 1 month, and achieving normal levels 14 months after CART19) and IgM (detectable at 1 month, with normal levels at 14 months). He was able to discontinue IgRT 10 months after CART19 loss, with no infections, maintaining normal IgG levels and developing specific IgG to *B. pertussis* after wild-type infection. He refused revaccination. Two years after CART loss he experienced an ALL relapse.

## Discussion

CART19 therapies have revolutionised the treatment of B-cell malignancies, but the short follow-up experience with this therapy precludes defining the degree of secondary immunodeficiency and recommendations for its management. Our descriptive study first analysed the kinetics of BCA and dysgammaglobulinemia in a paediatric cohort treated with two different CART19 constructs, exploring the possibility of an immune reservoir for immunoglobulin synthesis. And second, it offers a description of the long-term B-cell recovery of three patients after CART19 loss. The evidence obtained may contribute to modifying current IgRT and follow-up recommendations in children treated with CART19.

CAR19 T cells have the ability to recognise and destroy CD19-expressing cells of the B-lineage. Since normal B-cells are also eliminated, this ON target-OFF tumour effect causes a secondary progressive *dys*- or agammaglobulinemia, which lasts as long as CAR T cells remain functional. In adult patients, who may have a more complete antibody repertoire produced by pre-existing CD19-negative plasma cells, the indications for IgRT remain unclear [20], but the consensus seems to be to prescribe IgRT in case of recurrent or serious infections or when IgG < 4 g/L [14, 20]. In paediatric patients, early empiric IgRT after CAR T-cell infusion with trough levels >8–10 g/L is recommended [15] as long as CAR T cells persists, similar to what is indicated for patients with primary immunodeficiencies with absent B cells, such as X-linked agammaglobulinemia [22]. In this primary immunodeficiency, there is a congenital arrest of B-cell differentiation at the pre-B cell stage in the bone marrow, and affected patients, usually with an inborn severe decrease in mature B cells and consequently plasma cells, manifest with markedly decreased numbers of peripheral B cells (<1%) and consequently show very low or absent IgG, IgA and IgM [23]. This IgRT approach appears to mitigate most acute infectious complications [20]. This is the recommendation we followed, starting IgRT at day 28 after CART19 infusion. All of our patients maintained the recommended IgG trough levels during B-cell aplasia without presenting recurrent or severe infections after day 28 of CART19 infusion, except for a single patient with *H. influenzae* meningitis. This was a very young boy (11 months old at the ALL onset, 2.5 years at the time of CART19, and completely agammaglobulinemic after CART19 therapy), attesting to the safety of this recommendation. Despite the positive message regarding the safety of the IgRT recommendations, similar to those in agammaglobulinemic patients, it should be noted that in our cohort, 70% of treated patients progressed to undetectable IgM. But surprisingly, only 12% reached undetectable IgA levels. Thus, only 10% of the patients were completely agammaglobulinemic, suggesting the possible presence of a larger immune-reservoir than was previously thought, and raising other management questions for this population.

Since CART cell is a living drug because it is a cell, it can persist for a long time. This durability is beneficial for immunosurveillance of malignant cells, but it can entail a risk by reducing humoral immunocompetence while B-cell aplasia persists. This persistence differs depending on the CART19 products (type of construct, manufacture or subtype of cell), the targeted patient (adult/child), and the baseline disease (acute lymphoblastic leukaemia, chronic lymphocytic leukaemia, diffuse-large B-cell lymphoma other lymphoma types, etc.) [16]. In our cohort, which included only children with ALL but with two different CART19 constructs, at 24 months after CART19 infusion up to 60% remained with BCA (Fig. 2a), consistent with the observed long persistence of some 4-1BB CART19 constructs [1, 24].

The evaluation of the degree of secondary immunodeficiency and its associated infectious risk during the period of functional CART19 remains difficult. Indeed, plasma B-cells (PC) (long- and short-lived), which should be CD19 negative, might not be destroyed by CART19 therapy and may persist after treatment [16]. Plasma cells are clone-specific antibody factories. Protective antibodies are secreted by pre-existing plasma cells and reactivated antigen-experienced memory B-cells, and they constitute the main humoral immune defence (also called humoral memory response, as opposed to primary antibody response) [25]. CART19 eliminates memory B cells but may preserve plasma cells, enabling a certain immune competence in previously exposed individuals. This essential fact probably conditions the differences expected in the degree of immunodeficiency during CAR19 T-cell therapy between children and adults, since children will have a far more reduced plasma cell repertoire, specially under 10 years old [26]. In the absence of B cells, destroyed by CART19, the plasma cell reservoir is the only source of humoral immune defence, and it will condition, at least in part, our therapeutic decisions aimed at diminishing the infectious risk, including immunoglobulins and antibiotic prophylaxis. In fact, recent studies have assayed the use of direct plasma cells marker (BCMA) to evaluate the severity of the different forms of primary antibody deficiencies (PAD), concluding that a higher level of BCMA, which correlates with a good plasma cell reservoir, is indicative of a less severe form of PAD [13] with less infectious risk. Thus, the measurement of plasma cell reservoir could be crucial in the management of these patients.

Since plasma cells in humans are located in bone marrow (mainly IgG and IgA-long-lived PC) and secondary lymphoid organs (mainly in spleen but also in lymph nodes, where IgM-shorted-lived PCs are the main group of PCs), they are not measurable in peripheral blood, so only indirect measures using serum BCMA, as suggested by Cunningham et al. [13], might prove useful in quantifying this reservoir. In order to evaluate the plasma cell reservoir, some indirect markers determined in our patients could be helpful; immunoglobulin kinetics may be one of these surrogate markers. IgG is frequently not useful because of IgRT. As an alternative, levels of IgA and IgM can be studied. In our cohort, both immunoglobulins decreased rapidly after CART19 therapy: IgM tended to disappear (22 of 31 patients achieved undetectable IgM) during the 2 months following the infusion and IgA remained detectable in almost all patients (only 4 of 31 presented undetectable IgA). Different reasons could explain these observations. On the one hand, there is the disappearance of the PC source, the mature B-cell, due to prior B-cell targeted therapies or other chemotherapies, as well as CART19, together with the different half-life of the IgA-producing long-lived PCs (usually years) which seems to be longer than that of IgM-producing PCs (from weeks to months). Without this source, short-lived PCs are the first to disappear with a consequent hypo IgM. This effect has been described previously with the use of other B-cell targeted therapy such as Rituximab [27]. In our analysis, the use of prior B-cell targeted therapies such as (blinatumomab, rituximab, inotuzumab and bortezomib) was associated only with undetectable increased levels of IgA after CART19 but not with changes in IgM. This observation may be indicative of the impact on the PC reservoir for a longer period of time than the mere CART19. Nevertheless, it should be noted that these B-cell targeted therapies are usually part of advanced lines of treatment for refractory ALL, and thus the PC reservoir damage should not only be ascribed only to them: in fact, the median number of prior ALL lines of therapy in our cohort was 2 lines (see Table 1), but from the nine patients with prior B-cell targeted therapies, five had received more than two lines (two patients received five lines, one patient received four lines and two patients received three lines).

On the other hand, a possible direct effect of CART19 on PC should be considered. Although the lack of CD19 expression on PC is acknowledged, recent studies have been carried out on the use of CART19 therapy in multiple myeloma patients, achieving good responses. This has led to the final demonstration that a very low CD19 expression exists in some PCs by using single molecule-sensitive direct stochastic optical reconstructing microscopy [28]. Therefore, it would be advisable to reach a consensus whether there is sufficient evidence of PC persistence, at least for of IgA-PC in some paediatric patients after CART19 therapy.

The relevance of the persistence of IgA-PC goes beyond its mere demonstration. Indeed, the ability of these cells to produce IgA and secretory IgA could contribute to lessening the risk of infection by providing mucosal protection [29]. In fact, in X-linked agammaglobulinemia (which was the basis on which to decide the increase in IgG trough levels to 8 g/L as opposed to 6 g/L as in other humoral deficiencies [14, 22]), one of the reasons for the increased infectious risk is the absence of secretory IgA. In this sense, of our 12 evaluated patients, IgA in saliva was detectable in four, and in three of them it lay within protective ranges.

In summary, the fact that the studied patients were not completely agammaglobulinemic, the presence of IgA in almost 88% of the patients, the detectable protective levels of IgA in saliva in some, and the low level of serious infections in these patients (only one patient, who was one of the agammaglobulinemic patients after CART19) may be indicative of the presence of a preserved immune reservoir in some children despite CART19 (those with preserved IgA/IgM levels). This may be taken into account for IgRT and prophylaxis management, especially in older patients (at ALL onset and at CART19 infusion) with more plasma cell repertoire, and therefore closer to meeting the criteria for adult recommendations. But in the absence of more data supporting this hypothesis, conservative and safer recommendations should apply to paediatric patients.

Another aspect of CART19 therapy is the long-term immune impact once CART19 loses its function. It is known that in certain B-cell targeted therapies such as rituximab, B-cell reconstitution might take anywhere from 6 months to 2 years [30, 31]. Little has been reported in the literature regarding B-cell reconstitution after CAR T-cell loss, but some studies, mainly in adults, have reported defects in B-cell reconstitution, in various situations: permanent BCA despite the absence of detection of CART19 copies by PCR, suggesting there might be CAR T-cell persistence but below the limits of detection methods [7], and quantitative B-cell recovery up to several hundred cells per microliter, with impaired IgG production. Regarding the latter, no detailed data are available and no hypothesis has been proposed to date [14]. Of our three patients showing B-cell recovery, two of three presented some degree of B-cell defect at 9 and 27 months after CART19 loss: one with normal B-cell levels but no immunoglobulin production, and the other with B-cell lymphopenia and undetectable IgA. Both of them required IgRT, despite normal T-cell function. Of note, both of these patients were very young at ALL onset (5 months and 2.5 years, respectively), and one of them (P3) received inotuzumab as part of the therapy prior to CART19. Age at the time of the first ALL, the intensity of the treatment received prior to CAR T-cell therapy, including HSCT, the CART19 type/construct, the T-cell status/sub-type used to perform the CART therapy (CD8+, or CD4+, naïve or memory, etc.), and the persistence of the CAR T-cell may all play a role in this persistent defect.

In summary, during the functional CART19 period, immunoglobulin kinetics show a rapid decrease below the normal levels per age of both IgA and IgM. Moreover, undetectable IgM is observed in the majority of patients, with undetectable IgA occurring much less frequently; some patients show protective levels of secretory IgA, revealing a possible immune reservoir of IgA-plasma cells. After CART19 loss, and despite quantitative B-cell recovery and in the absence of T-cell-associated defect, there is evidence of B-cell dysfunction, as demonstrated by a low IgM and/or IgA as well as a defective response to *S. tiphy* vaccination, indicating that B-cell reconstitution might take longer than expected. This is the case with other B-cell depleting therapies, thereby hampering the withdrawal of IgRT. Further follow-up along with improved techniques to quantify minimal residual CART19 activity and assess the plasma cell reservoir are needed to better our understanding of the degree of secondary immunodeficiency and the mechanisms underlying the delayed B-cell reconstitution observed in a subgroup of patients.

## Supplementary information

supplementary information
