# Supplementary material for: Kinetics of humoral deficiency in CART19-treated children and young adults with acute lymphoblastic leukaemia
Source: Bone Marrow Transplant. 2020 Aug 14;56(2):376–86. doi: 10.1038/s41409-020-01027-6 (PMC7870804; doi:10.1038/s41409-020-01027-6)
Supplement: Supplementary file 1 — supplementary information [file 41409_2020_1027_MOESM1_ESM.docx]

**Supplementary Material.**

SUPPLEMENTARY TABLE 1. CART19 prophylaxis protocol in our institution

| **Prophylaxis treatment** |  |
| --- | --- |
| Immunoglobulin replacement | Start at 28^th^ day after CAR T-cell infusion, (0.5 g/kg every 4 weeks) during all B cell aplasia period.  Recommended trough levels > 8 g/l  Initial administration: intravenous  After 6 months: subcutaneous administration is offered to patient and family |
| Amoxicillin | If :  - Patient had received total body irradiation during previous hematopoietic stem cell transplantation  - Or had a moderate-to-severe GVHD |
| Acyclovir | The first 6 months after CART19 infusion |
| Trimethoprim-sulfamethoxazole | Until confirmation of CD4+ >200/mm^3^ and normal T-cell proliferative responses to mitogens. |

GVHD: graft versus host disease; CART19: CD19-Chimeric antigen receptor;

SUPPLEMENTARY TABLE 2. Definitions of acute lymphoblastic leukemia outcomes.

| **Complete remission** | Less than 5% bone marrow blasts, without circulating blasts, and no extramedullary disease sites (assessed by physical exam, central system fluid analysis, and imaging studies, when required, distinguishing complete remission and complete remission with incomplete blood cell recovery. |
| --- | --- |
| **Minimal residual disease** | Less than 0.01% bone marrow blasts as assessed by flow cytometry, |
| **Relapsed disease** | Reappearance of blasts in blood or bone marrow or in extramedullary site after a complete remission, |
| **Refractory disease** | Failure to attain the criteria needed for any response categories or relapse, |

SUPPLEMENTARY TABLE 3. Factors related to humoral immunodeficiency during CART19 therapy.

3A. Factors related to undetectable IgA.

|  | **Detectable**  **27/31** | **Undetectable**  **4/31** | **p** |
| --- | --- | --- | --- |
| **Female** | 9/27 (33.3%) | 4/4 (100%) | 0.012 |
| **CART19 construct ARI-001** | 5/27 (18.5%) | 4/4 (100%) | 0.001 |
| **Previous HSCT** | 22/27 (81.5%) | 3/4 (75%) | 0.759 |
| **Total body irradiation** | 2/27 (7.4%) | 1/4 (25%) | 0.267 |
| **Prior B-cell targeted therapy** | 5/27 (18.5%) | 3/4 (75%) | 0.016 |
| **CRS** | 18/27 (66.7%) | 3/4 (75%) | 0.739 |
| **CRS grade III/IV** | 6/18 (33.3%) | 1/3 (33.3%) | 1 |
| **Mortality** | 2/24 (8.3%) | 1/4 (25%) | 0.267 |
| **Relapse** | 6/27 (22.2%) | 1/4 (25%) | 0.901 |
| **Previous immunoglobulin alteration** | 16/26 (61.5%) | 3/4 (75%) | 0.603 |
| **Previous IgA alteration** | 3/26 (11.5%) | 2/4 (50%) | 0.055 |
| **Abnormal T-cell proliferative response to mitogens after CART** | 2/12 (16.7%) | 0/2 (0%) | 0.533 |
| **Age at ALL** | 50.1 (3.3-257.5) | 54.9 (18.7-133) | 0.976 |
| **Age at CART19 infusion** | 8.9 (2.9-24.8) | 10.1 (3.9-15.8) | 0.860 |
| **CART19 persistence** | 294 (15-1261) | 97.5 (57-371) | 0.346 |
| **Time between ALL diagnosis and CART19 infusion (months) (median, IQR)** | 54.4 (20.8-139.2) | 41.4 (29.4-114.6) | 0.746 |

ALL: acute lymphoblastic leukaemia; IQR: interquartile range; CART19: CD19-Chimeric antigen receptor; HSCT: haematopoietic stem cell transplant; CRS: cytokine release syndrome.

3B. Factors related to undetectable IgM.

|  | **Undetectable**  **22/31** | **Detectable**  **9/31** | **p** |
| --- | --- | --- | --- |
| **Female** | 10/22 (45.5%) | 3/9 (33.3%) | 0.535 |
| **CART construct ARI-001** | 7/22 (31.8%) | 2/9 (22.2%) | 0.593 |
| **Previous HSCT** | 18/22 (81.8%) | 7/9 (77.8%) | 0.796 |
| **Body irradiation** | 3/22 (13.6%) | 0/9 (0%) | 0.244 |
| **Prior B-cell targeted therapy** | 6/22 (27.3%) | 2/9 (22.2%) | 0.771 |
| **CRS** | 15/22 (68.2%) | 6/9 (66.7%) | 0.935 |
| **CRS grade III/IV** | 4/15 (26.7%) | 3/6 (33.3%) | 0.306 |
| **Mortality** | 2/22 (9%) | 1/9 (11.1%) | 0.863 |
| **Relapse** | 5/22 (22.7%) | 2/9 (22.2%) | 0.976 |
| **Previous immunoglobulin alteration** | 11/21 (52.4%) | 8/9 (88.9%) | 0.057 |
| **Abnormal T-cell proliferative response to mitogens after CART19** | 2/13 (15.4%) | 0/1 (0%) | 0.672 |
| **Previous IgM alteration** | 9/21 (42.8%) | 7/9 (77.8%) | 0.079 |
| **Age at ALL** | 42 (3.3-182.3) | 78.4 (15.7-257.5) | 0.117 |
| **Age at CART19** | 8.1 (2.9-24.9) | 11.1 (3.9-24.2) | 0.184 |
| **CART19 Persistence** | 373.5 (43-1261) | 60 (15-296) | 0.001 |
| **Time between ALL diagnosis and CART19 infusion (months) (median, IQR)** | 52.1 (20.8-139.2) | 43.4 (28.4-134.1) | 0.828 |

ALL: acute lymphoblastic leukaemia; IQR: interquartile range; CART19: CD19-Chimeric antigen receptor; HSCT: haematopoietic stem cell transplant; CRS: cytokine release syndrome.
